# CGRWDL: alignment-free phylogeny reconstruction method for viruses based on chaos game representation weighted by dynamical language model

**DOI:** 10.3389/fmicb.2024.1339156

**Published:** 2024-03-20

**Authors:** Ting Wang, Zu-Guo Yu, Jinyan Li

**Affiliations:** ^1^National Center for Applied Mathematics in Hunan, Xiangtan University, Xiangtan, Hunan, China; ^2^Key Laboratory of Intelligent Computing and Information Processing of Ministry of Education, Xiangtan University, Xiangtan, Hunan, China; ^3^School of Computer Science and Control Engineering, Shenzhen Institute of Advanced Technology, Shenzhen, Guangdong, China

**Keywords:** virus phylogeny reconstruction, alignment-free method, chaos game representation, dynamical language model, *k*-mers

## Abstract

Traditional alignment-based methods meet serious challenges in genome sequence comparison and phylogeny reconstruction due to their high computational complexity. Here, we propose a new alignment-free method to analyze the phylogenetic relationships (classification) among species. In our method, the dynamical language (DL) model and the chaos game representation (CGR) method are used to characterize the frequency information and the context information of *k*-mers in a sequence, respectively. Then for each DNA sequence or protein sequence in a dataset, our method converts the sequence into a feature vector that represents the sequence information based on CGR weighted by the DL model to infer phylogenetic relationships. We name our method CGRWDL. Its performance was tested on both DNA and protein sequences of 8 datasets of viruses to construct the phylogenetic trees. We compared the Robinson-Foulds (RF) distance between the phylogenetic tree constructed by CGRWDL and the reference tree by other advanced methods for each dataset. The results show that the phylogenetic trees constructed by CGRWDL can accurately classify the viruses, and the RF scores between the trees and the reference trees are smaller than that with other methods.

## 1 Introduction

Comparative analysis of biological sequences is one of the most fundamental aspect of bioinformatics. Through sequence comparison, differences between biological sequences can be identified, structural or functional information in biological sequences can be found, and then similarity and homology between sequences can be discovered. Sequence alignment is the traditional method for sequence comparison of biological sequences. The traditional methods for sequence comparison and phylogeny reconstruction rely on similarity analysis based on multiple sequence alignment (MSA). Some MSA based methods are widely used, such as ClustalW (Thompson et al., 1994), Muscle (Edgar, 2004), MAFFT (Katoh and Standley, 2013). Although the traditional MSA based approaches generally remain the references for sequence comparisons in phylogenetic analysis, the inherent computational complexity of MSA makes it incompatible with very large data sets available today (Bernard et al., 2019). The accuracy of the MSA based methods are limited not only by sequence identity, but also by multiple prior assumptions about the evolution of the sequences to be compared (Zielezinski et al., 2017). In addition, alignment algorithms usually assume that sequences of conserved regions are homologous segments of sequences (Li and Homer, 2010). However, this assumption often contradicts with real situations (Zielezinski et al., 2019). Therefore, in recent years, more and more research has focused on alignment-free approaches for phylogenetic analysis.

Many alignment-free methods for sequence comparison have been proposed, including information-theory-based methods (Out and Sayood, 2003; Li et al., 2004; Li and Vitányi, 2008; Giancarlo et al., 2014; Vinga, 2014; Bussi et al., 2021), which use information theories to estimate the amount of shared information between sequences and thus analyze the similarity of species. There are also alignment-free comparison methods based on Fourier transformations (Yin and Yau, 2015; Li et al., 2022), spaced-word (Leimeister et al., 2014a, 2017, 2019a,b; Morgenstern, 2021), iterated-function systems (Almeida, 2014), moments of the positions of the nucleotides (Li et al., 2016, 2017), common substring length (Ulitsky et al., 2006; Yang et al., 2012; Leimeister and Morgenstern, 2014; Yang W. F. et al., 2016), higher order Markov model and chaos game representation (CGR) (Yang L. et al., 2016) etc. In particular, CGR is an important method for phylogenetic analysis (Joseph and Sasikumar, 2006; Almeida, 2014; Sengupta et al., 2020; Sun et al., 2020; Löchel and Heider, 2021). Jeffrey (1990) proposed the CGR of DNA sequences. Due to the advantages of using CGR of DNA sequences, scholars started to study the CGR of protein sequences (Fiser and Tusnady, 1994; Basu et al., 1997; Yu et al., 2004a). Protein sequences are more complex compared to DNA sequences as they consist of 20 types of amino acids. Fiser and Tusnady (1994) proposed a CGR of protein sequences by mapping proteins into a positive 20-sided shape, with each of the 20 vertices representing an amino acid. Basu et al. (1997) generated CGRs for different protein families using positive 12-sides shape, where each vertex represents a set of amino acid residues based on conservative substitutions. Yu et al. (2004a,b) proposed a CGR of protein sequences based on a detailed HP (hydrophobic, polar) model. In such a model, the protein sequence is mapped into a 4-sides shape, and the 20 amino acids that constitute the protein sequence are divided into four categories according to the amino acid polarity, with each vertex representing an amino acid of one polarity. Multifractal analysis for the CGR of genomes is an alignment-free methodology that has been applied to study genomic variations between viral species (Pandit et al., 2012). In addition, methods based on substrings of fixed length (*k*-mer) are most widely used in the studies of phylogeny. The main idea is to extract some information from the *k*-mers of a biological sequence as the feature vector of that sequence, and then calculate the pairwise distance matrix between the feature vectors, and then use the distance matrix to construct a phylogenetic tree. The most used one is the frequency information of *k*-mers (Qi et al., 2004; Jun et al., 2010; Yu et al., 2010a,b; Sims and Kim, 2011; Luczak et al., 2019; Cattaneo et al., 2022). On the other hand, some scholars have done some analyses using the position information of *k*-mers (Kolekar et al., 2012; Xie et al., 2015; He et al., 2021; Wang et al., 2022; Tang et al., 2023). There is also the analysis that combines the frequency of *k*-mers as well as the position (Tang et al., 2021).

In this paper, we propose a new alignment-free method to construct the phylogenetic tree, which is named CGRWDL. We combine the dynamical language (DL) model and CGR to obtain new sequence information by considering both the frequency and context of *k*-mers (average position of *k*-mers) in the sequence. This combined consideration of the obtained information is used as a feature of the sequence to infer the phylogenetic tree. In detail, we use the DL model and CGR method to get the frequency information and the context information of the *k*-mers in the sequence, respectively. Then we consider the frequency and context of the *k*-mers together to extract more information from the *k*-mers in the sequence, so that the feature vector obtained will lose less information of the sequence, and the phylogenetic tree constructed will be closer to the reference tree.

From the feature vectors of *k*-mers in multiple sequences and their distance matrices, we performed phylogenetic analysis of DNA sequences and protein sequences. We constructed phylogenetic trees for eight datasets of viruses and compared them with the current state-of-the-art alignment-free methods demonstrate the superiority of our method in the accuracy of constructing phylogenetic trees.

## 2 Materials and methods

### 2.1 Datasets

To validate our metho hows the specific process of a sequence ATGC with o d, we applied our method for phylogeny reconstruction on complete DNA sequences, complete protein-coding DNA sequences and complete protein sequences of human immunodeficiency viruses (HIV-1), hepatitis C viruses (HCV), hepatitis B viruses (HBV), human rhinoviruses (HRV), human papillomaviruses (HPV), Dengue viruses, Ebola viruses, and Coronaviruses, respectively. The complete protein-coding DNA sequences are assembled from all coding sequences in the genome, and the complete protein sequences are assembled from all the protein sequences translated in this genome.

Human immunodeficiency virus (HIV) is a single-stranded RNA virus that can be divided into two types, HIV-1 and HIV-2. HIV-1 infection causes shorter disease duration, more severe symptoms, greater virulence, and greater threat to humans. HIV-1 (Lemey et al., 2004) can be divided into four subtype groups, M, N, O, and P, with 14 subtypes. The M subtype group includes 11 subtypes A, B, C, D, E, F, G, H, I, J, and K. The N subtype group and O subtype group contain only the N and O subtype, respectively. In these 14 subtypes, 13 subtypes are all previously discovered strains, while the P group of HIV-1 is the last HIV-1 type strain to be discovered, constituting only two strains so far. Dataset 1 in Supplementary material used here contains 56 HIV-1 viruses (Tang et al., 2023) of 11 subtypes A, B, C, D, F, G, H, J, N, O, and P. Among them, subtype A has two subtypes A1 and A2, and subtype F has two subtypes F1 and F2.

Hepatitis C virus (HCV) is a type of viral hepatitis virus, a single-stranded positive-stranded RNA virus. HCV viruses can be classified into types 1–6 (Chen and Morgan, 2006) according to the differences in gene sequences. Type 1 HCV is the most common and has distributed worldwide, predominant in China, the United States and Japan; type 2 HCV is common in China; type 3 HCV is common in India, China, Australia, and Pakistan; type 4 HCV is common in the Middle East and Africa; type 5 HCV is common in South Africa; and type 6 HCV is common in Hong Kong and Macau of China. He et al. (2020) used a dataset consisting of 82 HCV viral complete DNA sequences, but the complete protein-coding DNA sequences and complete protein sequences of 20 HCVs in this dataset are not available in NCBI (see details in the Supplementary material). Hence we only use the 62 HCVs which have all three data types, complete DNA sequence, complete protein-coding DNA sequence and complete protein sequence as our Dataset 2 in Supplementary material. There is no type 5 HCV in these 62 HCVs.

Human rhinovirus (HRV), the most common pathogen causing viral respiratory infections in humans, is also among the most serotyped viruses in humans. About half of all colds in adults are caused by rhinovirus infections. HRVs are currently classified into three subtypes A, B and C (Bochkov et al., 2011). The Dataset 3 in Supplementary material used here is composed of 113 HRVs with 3 outgroup Hepatitis E viruses (HEVs) (He et al., 2021). Among them, 113 HRVs belong to subtype A, subtype B and subtype C, while 3 HEVs as outgroups can test the validity of our method more effectively.

Hepatitis B virus (HBV) is a hepatophilic DNA virus, and its infection can lead to hepatitis B, liver fibrosis, liver cancer, and other related diseases. HBVs have several genotypes (Locarnini and Zoulim, 2010) of A-H. Genotypes B and C are predominant in China, with type C mainly distributed in the north, type B mainly in the south, and types A, D, and F in the west and minority regions. The Dataset 4 in Supplementary material we used consists of 121 hepatitis B viruses, and these viruses have eight genotypes A, B, C, D, E, F, G, and H. The accession numbers are provided in the Supplementary materials.

Human papillomavirus (HPV), a spherical DNA virus, is widespread in nature and uses humans as the sole host, causing a variety of warts and neoplastic diseases when infected. Up to present, more than 150 HPV subtypes have been isolated and identified (Akgül et al., 2006). We used a dataset consisting 326 HPV viruses from He et al. (2020) as our Dataset 5 in Supplementary material, which belongs to 12 subtypes, 6, 11, 16, 18, 31, 33, 35, 45, 52, 53, 58, and 66.

Dengue fever is an acute insect-borne disease caused by dengue virus, which is an RNA virus and one of the most widely spread mosquito-borne infections in the world, with four main subtypes, 1, 2, 3 and 4 (Ross, 2010). Dengue fever is widely distributed in tropical and subtropical regions, with the most serious epidemics in Southeast Asia, the Western Pacific region and the Americas. In China, it is mainly prevalent in Guangdong, Hainan, Fujian, Taiwan, Guangxi and Zhejiang and other southern regions. Dataset 6 in Supplementary material we used contains 330 dengue viruses (He et al., 2021) belonging to the four subtypes.

Ebola virus, which first appeared in 1976, is a rare but serious and often fatal disease that can be caused in humans. Ebola virus is transmitted to humans through wildlife and spreads through interpersonal transmission in humans. The average disease mortality rate for Ebola is approximately 50%. Ebola virus is a single-stranded negative-stranded RNA virus with a genome consisting of approximately 18,900 bases, and it has been determined that the genus Ebola virus can be divided into five subtypes, namely Ebola-Zaire virus (EBOV), Ebola-Sudan virus (SUDV), Ebola-Reston virus (RESTV), Ebola-Bendibugio virus (BDBV), and Taif Forest virus (TAFV) (Jacob et al., 2020). The different subtypes have different properties, with EBOV and SUDV being highly pathogenic and lethal to humans and non-human primates; RESTV is not pathogenic to humans and has lethal effects in non-human primates. The large outbreaks that occurred in West Africa from 2014 to 2016 were primarily caused by the Zaire-type Ebola virus. Our Dataset 7 in Supplementary material has 59 Ebola viruses (Das et al., 2020) belonging to five subtypes.

Coronaviruses are single-segmented positive-stranded RNA viruses, a large group of viruses that are widely found in nature. Coronaviruses were first isolated from chickens in 1937, and the family is the largest known family of RNA viruses, divided into four genera: α-viruses, β-viruses, γ-viruses, and δ-coronaviruses (Yang and Leibowitz, 2015). The Dataset 8 in Supplementary material we used contains 66 coronaviruses (Kirichenko et al., 2022), of which 57 viruses belong to the four genera α, β, γ, and δ, and 9 viruses are still unclassified so far. All of the sequences were taken from NCBI GenBank.

These 8 datasets have sequence lengths ranging from 3,248 to 29,821, and, these datasets (except Dataset 4 in Supplementary material) have been previously used by other scholars. Here, we use these datasets as references to evaluate our method by comparing its results with those obtained by other alignment-free methods Details of these datasets can be referred in the Supplementary material.

### 2.2 Methods

#### 2.2.1 Dynamical language model

The algorithm used in DLTree has been described in detail by Yu et al. (2005, 2010a,b). Let S = $s_{1}s_{2}\ldots s_{L}$ denote a DNA sequence (or protein sequence) with length *L*, where for any $i\in \left\{1,2,\ldots ,L\right\}$ , $a_{i}\in \left\{A,C,G,T\right\}$ (or {A, I, L, M, F, P, W, V, D, E, N, C, Q, G, S, T, Y, R, H, K}), $a_{1}a_{2}\ldots a_{k}$ is a *k*-mer. First, we count the number of times of this *k*-mer in this sequence, denoted as *N*( $a_{1}a_{2}\ldots a_{k}$ ), and then calculate the frequency *P*( $a_{1}a_{2}\ldots a_{k}$ ) of this *k*-mer in this sequence. *P*( $a_{1}a_{2}\ldots a_{k}$ ) is defined as:


(1) $$P\left(a_{1}a_{2}\ldots a_{k}\right)=\frac{N\left(a_{1}a_{2}\ldots a_{k}\right)}{L-K+1}$$

Yu et al. (2005) used the theory of dynamical language to construct the expected frequency *Q*( $a_{1}a_{2}\ldots a_{k}$ ) of *k*-mer $a_{1}a_{2}\ldots a_{k}$ as:


(2) $$Q\left(a_{1}a_{2}\ldots a_{k}\right)=\frac{P\left(a_{1}\right)P\left(a_{2}a_{3}\ldots a_{k}\right)+P\left(a_{1}a_{2}\ldots a_{k-1}\right)P\left(a_{k}\right)}{2}$$

The relative deviation between *P*( $a_{1}a_{2}\ldots a_{k}$ ) (Eq. 1) and *Q*( $a_{1}a_{2}\ldots a_{k}$ ) (Eq. 2) is used to remove the background noise.


(3) $$M\left(a_{1}a_{2}\ldots a_{k}\right)=\frac{P\left(a_{1}a_{2}\ldots a_{k}\right)-Q\left(a_{1}a_{2}\ldots a_{k}\right)}{Q\left(a_{1}a_{2}\ldots a_{k}\right)}$$

We consider *M*( $a_{1}a_{2}\ldots a_{k}$ ) as the first feature extracted from the sequence.

#### 2.2.2 Chaos game representation

CGR of DNA sequence was proposed by Jeffrey (1990), which is an iterative function-based graphical representation of DNA sequences. CGR expresses the distribution rule of DNA sequences of a certain length as fractal characteristics of a graph, and then the distribution rule of the sequence can be obtained by fractal analysis. Therefore, it has become a statistical method for genome sequence analysis. CGR has become a powerful tool for feature encoding in machine learning and alignment-free sequence comparison (Löchel and Heider, 2021).

##### 2.2.2.1 Chaos game representation of DNA sequences

Each nucleotide of a DNA sequence is mapped one-to-one in order onto the unit plane, and the four vertices of the plane are the four nucleotides that make up the DNA sequence, where each base is located at the coordinates of: $P_{A}=\left(0,0\right)$ , $P_{C}=\left(0,1\right)$ , $P_{G}=\left(1,1\right)$ , $P_{T}=\left(1,0\right)$ . And, the CGR can be represented by the following iterated function system (IFS):


(4) $$CGR_{i}=0.5\times \left(CGR_{i-1}+\omega_{i}\right)\text{,}$$

where, $CGR_{0}=\left(0.5,0.5\right),\omega_{i}=\{\begin{array}{c} \left(0,0\right),\mathrm{if}\;\omega_{i}\;\mathrm{is}\;A\\ \left(0,1\right),\mathrm{if}\;\omega_{i}\;\mathrm{is}\;C\\ \left(1,1\right),\mathrm{if}\;\omega_{i}\;\mathrm{is}\;G\\ \left(1,0\right),\mathrm{if}\;\omega_{i}\;\mathrm{is}\;T \end{array},\;i\in$ {*1,2,…,L*}.

**Figure 1:**
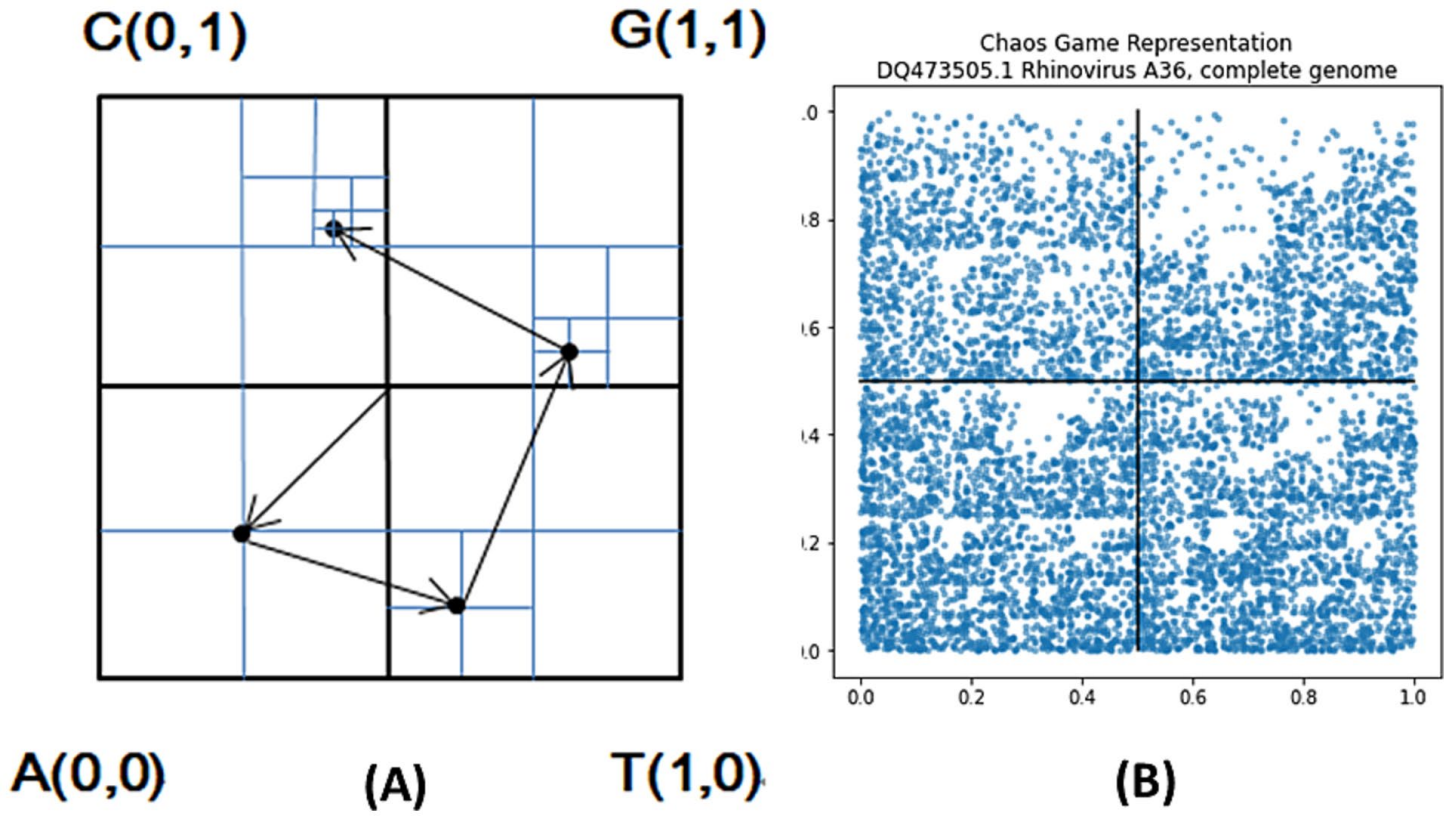
**(A)** CGR generation process (e.g., ATGC), **(B)** CGR of complete DNA sequence of HRV: DQ473505.1.

The Figure 1A shows the specific process of a sequence ATGC with only 4 bases mapped point-by-point to the unit plane (CGR plot generation), and the Figure 1B is a CGR plot corresponding to complete DNA sequences of HRV.

##### 2.2.2.2 Chaos game representation of protein sequences

Since protein sequences are more complex than DNA sequences and consist of 20 amino acids, we refer to the CGR of protein sequences based on the detailed HP model proposed by Yu et al. (2004a) in order to have some correspondence with the CGR representation of DNA sequences.

Similar to the CGR of DNA sequence, each amino acid in a protein sequence is mapped to the unit plane in a one-to-one order. Now the four vertices of the unit plane are the 20 amino acids that make up the protein sequence instead of four nucleotides that make up the DNA sequence. Here, we classify amino acids into four categories according to their polarity (Yu et al., 2004b), namely: non-polar class: A, I, L, M, F, P, W, V; negative polar class: D, E; uncharged polar class: N, C, Q, G, S, T, Y; and positive polar class: R, H, K. where the coordinates of the position where each amino acid is located are: $P_{\beta }=\left(0,0\right)\text{,}$ if β = A, I, L, M, F, P, W, or V; $P_{\beta }=\left(0,1\right)\text{,}$ if β = D or E; $P_{\beta }=\left(1,0\right)\text{,}$ if β = R, H or K; $P_{\beta }=\left(1,1\right)$ , if β = N, C, Q, G, S, T or Y.

Its iterative function system is expressed as:


(5) $$CGR_{i}=0.5\times \left(CGR_{i-1}+\varphi_{i}\right)\text{,}$$

where, $CGR_{0}=\left(0.5,0.5\right),\varphi_{i}=\{\begin{array}{l} \left(0,0\right),\mathrm{if}\;\varphi_{i}\;\mathrm{is}\;A,I,L,M,F,P,W,V\\ \left(0,1\right),\mathrm{if}\;\varphi_{i}\;\mathrm{is}\;D,E\\ \left(1,0\right),\mathrm{if}\;\varphi_{i}\;\mathrm{is}\;R,H,K\\ \left(1,1\right),\mathrm{if}\;\varphi_{i}\;\mathrm{is}\;N,C,Q,G,S,T,Y \end{array}\;,\;i\in$ {*1,2,…,L*}.

##### 2.2.2.3 Position mean of chaos game representation

Whether it is a DNA sequence or a protein sequence, for each *k*-mer, we can find the prefix part of this sequence ending with that *k*-mer and can also get the CGR of this subsequence. We denote the last position of the subsequence ending with a *k*-mer $a_{1}a_{2}\ldots a_{k}$ in the CGR diagram as ( $CGR_{a_{1}a_{2}\ldots a_{k}}x,\;CGR_{a_{1}a_{2}\ldots a_{k}}y$ ). In a sequence, a *k*-mer may appear several times, so there are several subsequences ending with that *k*-mer, hence there may have multiple coordinate values in the CGR graph. And we calculate the position means $CGR_{a_{1}a_{2}\ldots a_{k}}\bar{x}$ and $CGR_{a_{1}a_{2}\ldots a_{k}}\bar{y}$ for these coordinate values.


(6) $$\begin{array}{l} \;CGR_{a_{1}a_{2}\ldots a_{k}}\bar{x}=\\ \frac{\left(CGR_{a_{1}a_{2}\ldots a_{k}}x_{1}+CGR_{a_{1}a_{2}\ldots a_{k}}x_{2}\cdots +CGR_{a_{1}a_{2}\ldots a_{k}}x_{N_{A}}\right)}{N_{A}}\text{,} \end{array}$$

(7) $$\begin{array}{l} CGR_{a_{1}a_{2}\ldots a_{k}}\bar{y}=\\ \frac{\left(CGR_{a_{1}a_{2}\ldots a_{k}}y_{1}+CGR_{a_{1}a_{2}\ldots a_{k}}y_{2}\cdots +CGR_{a_{1}a_{2}\ldots a_{k}}y_{N_{A}}\right)}{N_{A}}\text{,} \end{array}$$

where $N_{A}=n\left(a_{1}a_{2}\ldots a_{k}\right)$ , is the number of times *k*-mer $a_{1}a_{2}\ldots a_{k}$ appears in this sequence.

We consider $CGR_{a_{1}a_{2}\ldots a_{k}}\bar{x}$ and $CGR_{a_{1}a_{2}\ldots a_{k}}\bar{y}$ as the second feature extracted from the sequence. An example to show calculating process is given in Supplementary material. We mapped the sequence to a CGR graph, where each nucleotide in the sequence corresponds to a point in a unit square based on the position of this nucleotide in the sequence. Each point has a coordinate, and this coordinate value represents the position of the subsequence ending with this *k*-mer in the CGR graph (this corresponds the position of this *k*-mer in the sequence). We know that in a sequence, the same *k*-mer may appear multiple times at different positions in the sequence, hence we use the average of the CGR coordinate values of this *k*-mer to represent the average position of this *k*-mer in the sequence. In fact, the average position here indicates the context information of *k*-mer.

#### 2.2.3 Chaos game representation weighted by dynamical language model

In the previous subsection, we obtained $M\left(a_{1}a_{2}\ldots a_{k}\right)$ using the DL model, and we use $M\left(a_{1}a_{2}\ldots a_{k}\right)$ (Eq. 3) as the weight value of the corresponding CGR region mean $CGR_{a_{1}a_{2}\ldots a_{k}}\bar{x}$ (Eq. 6) and $CGR_{a_{1}a_{2}\ldots a_{k}}\bar{y}$ , (Eq. 7) weighting $CGR_{a_{1}a_{2}\ldots a_{k}}\bar{x}$ and $CGR_{a_{1}a_{2}\ldots a_{k}}\bar{y}$ with $M\left(a_{1}a_{2}\ldots a_{k}\right)$ to obtain:


$$\begin{array}{l} S_{i}\left(a_{1}a_{2}\ldots a_{k}\right)=\\ \left(M\left(a_{1}a_{2}\ldots a_{k}\right)\times CGR_{a_{1}a_{2}\ldots a_{k}}\bar{x},M\left(a_{1}a_{2}\ldots a_{k}\right)\times CGR_{a_{1}a_{2}\ldots a_{k}}\bar{y}\right) \end{array}$$

We denote $M\left(a_{1}a_{2}\ldots a_{k}\right)\times CGR_{a_{1}a_{2}\ldots a_{k}}\bar{x}$ as *Info_X* and $M\left(a_{1}a_{2}\ldots a_{k}\right)\times CGR_{a_{1}a_{2}\ldots a_{k}}\bar{y}$ as *Info_Y*. In particular, *Info_X* and *Info_Y* are 0 when *k*-mer $a_{1}a_{2}\ldots a_{k}$ is not occurring. For species A, for a fixed *k*-value, there are $4^{k}$ different *k*-mers, hence we can obtain $4^{k}$ *Info_X* and *Info_Y*. We arrange these $4^{k}$ Info_X and *Info_Y* according to the alphabetical order of *k*-mers to obtain a $2\times 4^{k}$ -dimensional (or $2\times 20^{k}$ -dimensional) feature vector A. The first $4^{k}$ dimensions of this vector are *Info_X*, and the last $4^{k}$ dimensions are *Info_Y*, expressed as follows:


$$A=\left(S_{A,1}\;\cdots \;S_{A,4^{k}},\begin{array}{ccc} S_{A,4^{k}+1} & \cdots & S_{A,2\times 4^{k}} \end{array}\right)\text{.}$$

Each of the DNA sequences (or protein sequences) was mapped to a feature vector, an $n\times \left(2\times 4^{k}\right)$ *-*sized feature matrix can be obtained for *n* sequences as follows:


$$\left[\begin{array}{cc} \begin{array}{ccc} S_{1,1} & \cdots & S_{1,4^{k}} \end{array} & \begin{array}{ccc} S_{1,4^{k}+1} & \cdots & S_{1,2\times 4^{k}} \end{array}\\ \begin{array}{ccc} \vdots & \ddots & \vdots \\ S_{n,1} & \cdots & S_{n,4^{k}} \end{array} & \begin{array}{ccc} \vdots & \ddots & \vdots \\ S_{n,4^{k}+1} & \ldots & S_{n,2\times 4^{k}} \end{array} \end{array}\right]\text{.}$$

#### 2.2.4 Distance calculation

We take the definition of Manhattan distance to calculate the distance between two species. The distance between species A and species B is given by:


(8) $$d\left(A,B\right)=\overset{\gamma }{\underset{i=1}{\sum }}\left|S_{A,i}-S_{B,i}\right|\text{,}$$

where,


$$\gamma =\{\begin{array}{c} 2\times 4^{k},DNA\;\mathrm{sequences}\\ \;2\times 20^{k},\mathrm{protein}\;\mathrm{sequences} \end{array}\text{.}$$

After calculating all pairwise distances, a distance matrix D can be obtained, which reflects the differences between sequences or species. Finally, the Neighbor-Joining (Saitou and Nei, 1987) algorithm [50] is applied to construct a phylogenetic tree using MEGA X software (Kumar et al., 2018).

### 2.3 How to estimate the optimal length of *k*-mer

Wu et al. (2009) gave the definition of the cumulative relative entropy (CRE) and relative serial divergence in the feature frequency profile (FFP) method to estimate the optimal *k*-value. Similar to the idea of CRE and cumulative average relative deviation (CARD) (Xie et al., 2015), here we propose *cumulative average feature value* (CAFV) (defined below) to estimate the optimal *k*-value for our method. In the previous section 2.2.3, it is known that for species A, a feature vector $\left(s_{A,1},s_{A,2}\cdots s_{A,2\times 4^{k}}\right)$ can be obtained. First, we calculate the mean value of the feature vector, denoted as $V_{A,k}$ :


(9) $$V_{A,k}=\frac{\sum_{i}^{2\times 4^{k}}S_{A,i}}{2\times 4^{k}}$$

Then for *n* species, we can obtain a *V* (Eq. 9) for each of the *n* sequences and then sum the different *Vs* for the *n* species:


(10) $$T_{k}=V_{1,k}+V_{2,k}+\cdots +V_{n,k}\text{,}$$

where, *n* is the number of sequences.

Finally, the $T_{k}$ value (Eq. 10) obtained by taking different values of *k* and accumulating them in increasing order of *k* gives:


(11) $$CAFV_{k}=\overset{k}{\underset{1}{\sum }}\;T_{k}$$

*CAFV* value is calculated based on the feature vector taken from each sequence. When the length of *k*-mer is short, the information of the original sequence contained in our calculated feature vectors increases with the length of the *k*-mer, so the CAFV value also grows rapidly. After *k* reaches the optimal value, the information starts to decrease when *k* is increased (due to the fact that when the value of *k* is too large, many *k*-mers do not appear in the sequence, hence there are lots of zeros in the feature vectors, making the calculated CAFV value small). After the optimal value, the CAFV value starts to increase very slowly.

### 2.4 Robustness test

In order to test the robustness of the phylogenetic tree constructed using our method, we used the modified bootstrap method proposed by Yu et al. (2010a). The works as follows. We first use CGRWDL to extract sequence information to construct a feature matrix, where each row represents the feature vector of a DNA sequence (or protein sequence) and each column is the feature value of each fixed *k*-mer in different sequences. Afterwards, we randomly sample this $n\times \left(2\times 4^{k}\right)$ -dimensional (or $n\times \left(2\times 20^{k}\right)$ -dimensional) feature matrix by column with replacement. Then, we can obtain a new $n\times \left(2\times 4^{k}\right)$ -dimensional (or $n\times \left(2\times 20^{k}\right)$ -dimensional) matrix by sampling $n\times \left(2\times 4^{k}\right)\left(\mathrm{or}\;n\times \left(2\times 20^{k}\right)\right)$ times. In the next part, we use Equation (8) to calculate the Manhattan distance between every two rows in the new matrix to obtain the new distance matrix. After that, the method in Section 2.2.4 is used to construct the phylogenetic tree. In the end, keep repeating this process 100 times. Furthermore, we employed the method for estimating tree inconsistency based on information theory proposed by Salichos et al. (2014) to construct the inconsistency trees for each dataset, in which internode certainty (IC) and IC All (ICA) values were displayed on the branches of the trees.

## 3 Results

To demonstrate that our method is effective for phylogenetic analysis of virus sequences, we did the analysis on three types of data including complete DNA sequences, complete protein-coding DNA sequences and complete protein sequences from eight datasets of viruses HIVs, HCVs, HRVs, HBVs, HPVs, dengue viruses, Ebola viruses and coronaviruses.

### 3.1 Selection of *k*-value

**Figure 2:**
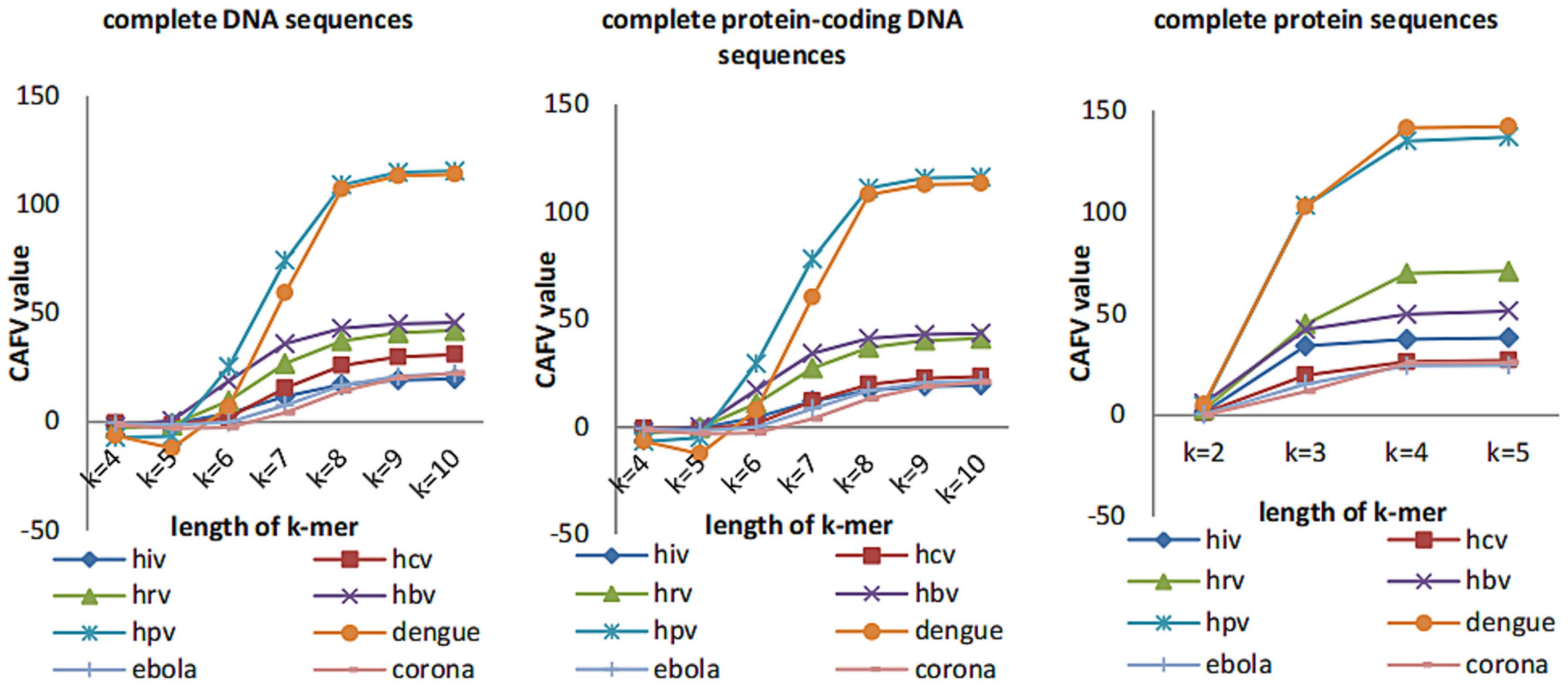
The CAFV values of the eight datasets change with *k*-mers length.

We plot the CAFV values (calculated by (Eq. 11)) for *k* from 4 to 10 for both DNA and protein sequences of 8 datasets of viruses in Figure 2. We can see that CAFV values tend to be stable when *k*-value is greater than or equal to 8 for complete DNA sequences and complete protein-coding DNA sequences, CAFV values tend to be stable when *k*-value is equal to 4 for complete protein sequences. Therefore, we guess *k*-value can be taken as 8 for complete DNA sequences and complete protein-coding DNA sequences and *k*-value can be taken as 4 for protein sequences. However, for longer sequences (such as coronaviruses), the CAFV value is still increasing slowly when *k*-value is changed to 10 for complete DNA sequences and complete protein-coding DNA sequences. In this case, we guess to set the value of *k* to 11.

In order to analyze the effect of the length of the *k*-mers on the performance of the CGRWDL, we tested it on datasets of different types and different lengths. We evaluated the performance of CGRWDL in complete DNA sequences, complete protein-coding DNA sequences and complete protein sequences, respectively. For each dataset, we used the phylogenetic tree constructed by alignment tool MUSCLE (Edgar, 2004), the Maximum Likelihood method and Tamura-Nei model (Koichiro and Masatoshi, 1993) in the Mega X software (used the default parameters) (Kumar et al., 2018) as the reference tree.

**Figure 3:**
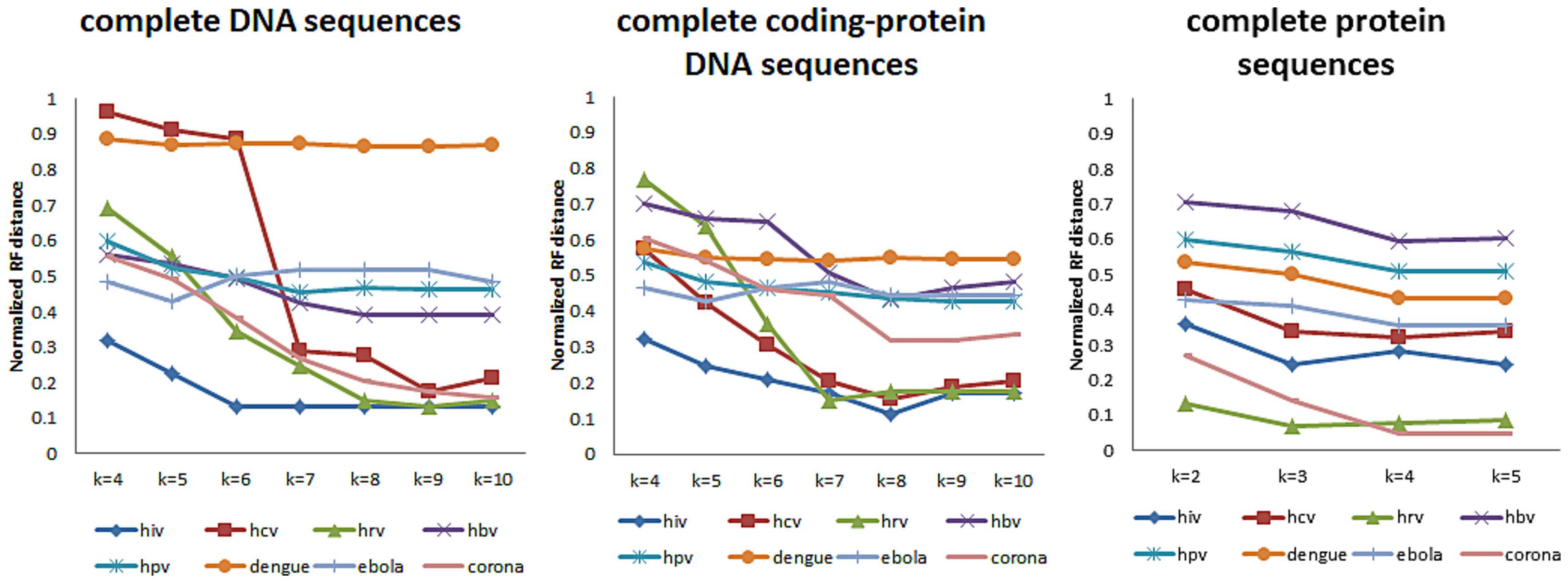
When *k*-mers takes different values, the Normalized RF distance between the phylogenetic tree constructed and the reference tree of the eight datasets.

Then we use the *treedist* in phylip (Felsenstein, 2005) to calculate the Robinson-Foulds (RF) distance (Robinson and Foulds, 1981) between the phylogenetic tree constructed by our method and the reference tree. Based on the variation of the RF distance, it is possible to see how *k*-value affects the results on different types and lengths of datasets. Figure 3 shows the RF distance between the phylogenetic tree generated by our method and the reference tree when *k* takes different values.

In Figure 3, we tested k-values between 4 and 10 when the data type is DNA sequences (both complete DNA sequences and complete protein-coding DNA sequences) and between 2 and 5 when the data type is protein sequences. And, the trend of RF distance between the phylogenetic tree constructed by CGRWDL and the reference tree tend to be stable when *k*-value is greater than or equal to 8 for DNA sequences. At the same time, the RF values of most sequences are minimized. For protein sequences, RF values are minimum values when *k*-value is equal to 4. But for viruses with longer sequences (such as Coronaviruses), it is required *k* = 10 for DNA sequences to obtain the minimum RF distance. This result is consistent with the conclusion that we use the CAFV value to estimate the optimal value of *k*. The *k*-value varies slightly with the length and type of viruses.

### 3.2 Phylogenetic analysis

**Figure 4:**
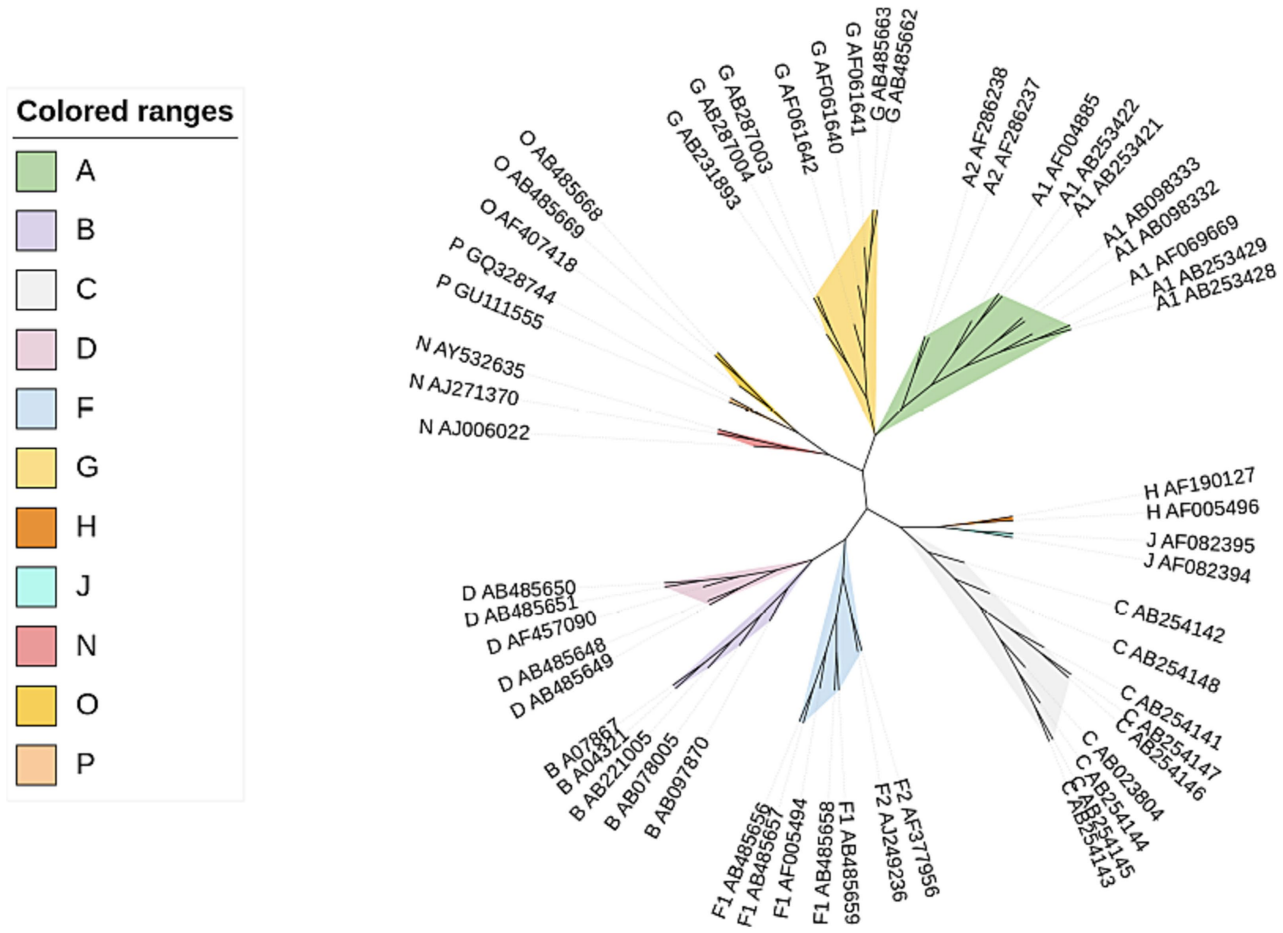
Phylogenetic tree of HIV-1 complete protein-coding DNA sequences constructed by CGRWDL (*k* = 8).

Here we used CGRWDL to construct the phylogenetic tree of 56 HIV-1 strains in Dataset 1 in Supplementary material, and the best phylogenetic tree was obtained when *k* = 8 for both the complete DNA sequences and complete protein-coding DNA sequences; while the best phylogenetic tree (the one with the smallest RF distance to the reference tree) was obtained when *k* = 3 for the complete protein sequences. We show the phylogenetic tree from the complete protein-coding DNA sequences as in Figure 4. We can see that the four subtype groups of HIV-1 genome sequences are clearly clustered and that the eight subtypes in group M are correctly classified. Two of the subtypes, A and F, have the correct subtype classification.

The phylogenetic tree from the complete protein-coding DNA sequences (Supplementary Figure S1) clearly clusters the HCV sequences of Dataset 2 in Supplementary material into five classes and all the five genotypes are correct, as shown in the five major branches of the tree.

**Figure 5:**
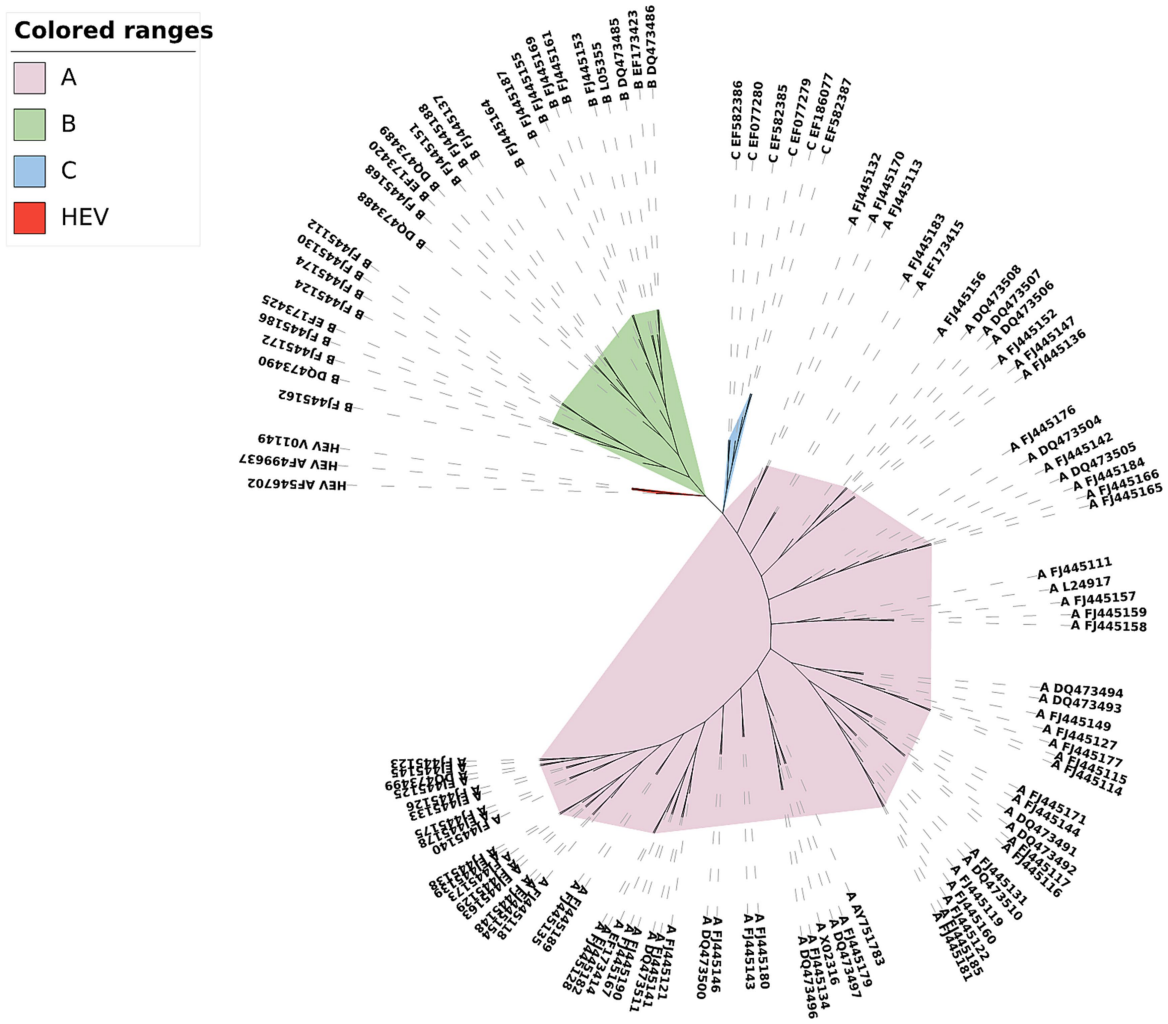
Phylogenetic tree of HRV complete protein sequences constructed by CGRWDL (*k* = 4).

Among the phylogenetic trees constructed for the three data types of HRVs in Dataset 3 in Supplementary material, the phylogenetic tree constructed for the complete protein sequences is the best of the three trees (see Figure 5). The three subtypes of the HRV sequences are clearly distinguished from each other, while the three HEV sequences as the outgroup are clustered together separately and are grouped into the outermost layer.

For HBV viruses in Dataset 4 in Supplementary material, we built three optimal phylogenetic trees for each of the three types of data when *k* is set 8 and 4, respectively. The most optimal phylogenetic tree was obtained from the complete DNA sequences (Supplementary Figure S2), where the HBV sequences were clearly divided into eight categories and each category contains only one subtype of HBV.

For human papillomaviruses in Dataset 5 in Supplementary material, among the phylogenetic trees we built using complete DNA sequences, complete protein-coding DNA sequences and complete protein sequences, the best performing one is the one constructed from complete DNA sequences with *k* = 8. As one can see in the figure (Supplementary Figure S3), the phylogenetic tree we constructed demonstrates that 330 HPV viruses are clustered into 12 clusters, each cluster corresponds to one subtype, and each sequence is clustered into the correct cluster.

For dengue viruses in Dataset 6 in Supplementary material, we constructed a phylogenetic tree by taking *k* = 8 for complete DNA sequences; a phylogenetic tree by taking *k* = 8 for complete protein-coding DNA sequences; and a phylogenetic tree with *k* = 4 for complete protein sequences. The best performance is the phylogenetic tree constructed by complete DNA sequences, as shown in Supplementary Figure S4. The figure shows the classification of the four subtypes. The dengue viruses were clustered into 4 classes and each class was correctly clustered.

To understand the relationship between Zaire-type viruses and other viruses, we used CGRWDL to construct a phylogenetic tree for Dataset 7 in Supplementary material. As shown in Supplementary Figure S5, the five Ebola genera are completely distinguished from each other.

**Figure 6:**
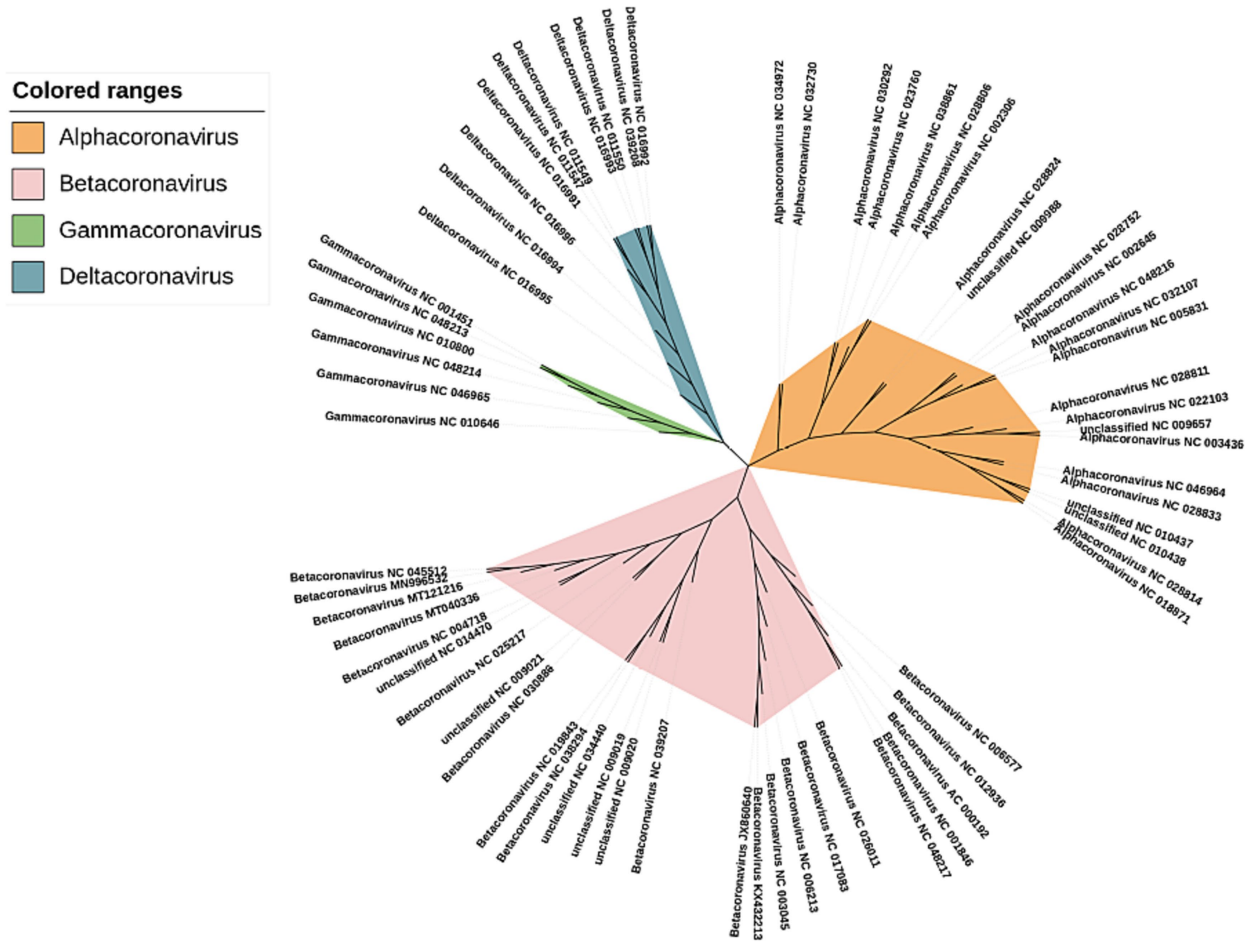
Phylogenetic tree of Coronaviruses complete DNA sequences constructed by CGRWDL (*k* = 10).

The phylogenetic trees were constructed using our method for the 66 sequences of the coronaviruses in Dataset 8 in Supplementary material (shown in Figure 6). We can see that these coronaviruses are clearly clustered into 4 categories α, β, γ, and δ. The previously unclassified NC_009657, NC_009988, NC_010437, NC_010438 are classified in the α cluster; NC_009019, NC_009020, NC_009021, NC_014470, and NC_034440 were classified to the β cluster.

### 3.3 The consistency analysis of trees

We conducted a consistency analysis on the phylogenetic trees inferred from the eight datasets using the CGRWDL method. We constructed inconsistency trees for each dataset, in which internode certainty (IC) and IC All (ICA) values (Salichos et al., 2014) were displayed on the branches of the trees. The inconsistency trees for all datasets are shown in the Supplementary material (Supplementary Figures S6–S13). From these figures, it can be seen that the IC and ICA values of 1 for each subtype branch in each dataset. However, there is some inconsistency at the fine branches within the same subtypes.

### 3.4 Performance comparison of CGRWDL with other methods

To compare the performance of our method with other advanced alignment-free methods, we used the same eight datasets mentioned above. Six state-of-the-art methods, namely DLTree (Yu et al., 2010a), CVTree (Qi et al., 2004), KINN (Tang et al., 2023), FSWM (Leimeister et al., 2017), FFP (Sims and Kim, 2011), and d2 (Vinga and Almeida, 2003) were used in the comparison. We note that the FSWM method can only do phylogenetic analysis for DNA sequences, while the other five methods are applicable to both DNA sequences and protein sequences. All six methods are based on *k*-mer techniques, among which the CV method utilizes Markov model; the DL method employs dynamic language model; the FFP method calculates distance scores based on the differences in *k*-mer frequencies between sequences; the d2 method computes distances based on *k*-mer frequencies using weighted distance; The KINN method proposes a new definition of *k*-mer inner distance; and the FSWM method is based on Spaced Word Matches.

We used all six methods to construct phylogenetic trees based on the eight datasets separately. We then calculated the RF distance between every phylogenetic tree and its reference tree, and the results are presented in Table 1. We mark the minimum score of the RF distance from the reference tree in each dataset in red, and mark the method that misclassified this dataset as “*.” Due to the fact that FSWM is only applicable to DNA sequences, a “/” is used to represent the column for the two protein sequences (Ebolaviruses (P), and HRV (P)) in the table. The *k*-mer values used for the seven methods are shown in Supplementary Table S1. Specifically, We constructed phylogenetic trees for different k values for each method separately and calculated the RF distance between these phylogenetic trees and the reference tree. The k-value corresponding to the smallest RF value was taken as the best k-value for the method, and is shown in Supplementary Table S1.

**Table 1:** RF distance comparison between phylogenetic trees constructed using 7 alignment-free methods and reference trees.

Dataset	CGRWDL	DLTree	CVTree	FFP	d2	KINN	FSWM
HIV	10	12	16	24	20	14	22
HCV	20	26	28	44	22	38	32
HBV	92	116	106	112	96	94	88
HPV	294	320	328	310	302	328	310
Dengue viruses	566	572	570	574	568	566	576
Coronavirus	20	20	38(*)	70(*)	38(*)	78(*)	30
Ebolaviruses(P)	40	50	54	50	52	50	/
HRV(P)	18	28	24	114	52	30	/

The minimum RF distance value for each virus indicated in red.

With these results shown in the table, we can see that CGRWDL is superior. In these eight datasets, the phylogenetic trees generated by our method have the smallest RF distances between the reference trees on the seven datasets. Only for HBV sequences our method does not have the smallest RF distance, but it is very close to the optimal method, with RF values differing by only 4 and ranking 2nd among the seven methods. As seen in the previous section that the groupings of the phylogenetic trees constructed using our method are all correct, so the RF distance values between the phylogenetic trees constructed by our method and the reference tree only reflect the topological differences between the internal branches of the same class on the phylogenetic tree generated by CGRWDL and the corresponding branches on the reference tree.

As can be seen from table, for HIV, the RF distance value obtained by our method is 10. After we analyzed the phylogenetic tree of HIV, we found that the difference between the phylogenetic tree of HIV constructed by our method and the reference tree only lies in small differences in the branch placement of the same genus species. In particular, the FFP, d2, CV, and KINN methods all made subtype classification errors when constructing phylogenetic trees for coronaviruses. Among them, the CV method classified the γ-viruses NC010646 virus in α-viruses; the d2 method divided the β-viruses into two parts; and the phylogenetic trees of the FFP and KINN methods mixed all the subtypes together. With correct categorization, the RF value of our constructed tree and the reference tree is smaller than FSWM by 16; the RF values of our constructed tree and the reference tree are equal to the DL method, both being 20.

## 4 Discussion

By comparing the performance of our method with other six state-of-the-art alignment-free methods, we can see that CGRWDL is superior. In most alignment-free methods, researchers represent DNA or protein sequences as numerical feature vectors that can describe the sequences, and use the similarity or dissimilarity between these feature vectors to describe the similarity or dissimilarity between sequences, thereby to build the phylogenetic tree. However, in the process of representing sequences as numerical feature vectors, some information is lost. The more information we used, the better the original sequence can be represented, and the more accurate the inferred phylogenetic tree can be. In this work, we propose the CGRWDL method, which combines the context and frequency information of *k*-mers. The context information is characterized by the average value in the CGR. The frequency information comes from the dynamic language model, which removes background noise. Therefore, our method used more information, allow us to infer phylogenetic tree more accurate.

Our method is an alignment-free method based on frequency and context information of *k*-mers. Similar to other alignment-free methods, the computational complexity of our method is not high. Therefore, our method is also applicable to large-scale sequence data.

As we all know, when *k*-mer is used for phylogenetic relationship analysis, the value of *k* is an important parameter that has a great influence on the results. How the length of *k*-mer should be taken is a topic that has always been discussed by scholars. Here we also discuss the selection of value of *k* in our method. From Section 3.1, it can be seen that the value of *k* changes with the length of the sequence. When the sequence is short, most of the time, *k* can be set to 8. However, if the sequence is longer, such as in the case of Ebola and Coronavirus, the value of *k* needs to be set to 10. Although the CGRWDL presented in this paper was used for viruses sequence comparison and phylogenetic tree reconstruction, it can also be used to analyze problems other than virus genome comparison such as bacterial genome comparison.

## 5 Conclusion

We proposed a new alignment-free comparison method called CGRWDL for viruses. The method is to combine the frequency information and context information of the *k*-mers in the sequence to obtain a new metric of the *k*-mers such that the sequence can be represented by this new *k*-mers metric. For different lengths and types of sequences, CGRWDL can accurately construct the phylogenetic relationships of species and the RF distance between it and the reference tree is smaller than other advanced methods. We also give a reference for the selection of the length of *k*-mers, and there is a slight difference in the length of *k*-mers to be selected for different lengths and types of datasets.

## Data availability statement

The original contributions presented in the study are included in the article/Supplementary material, further inquiries can be directed to the corresponding author.

## Author contributions

TW: Conceptualization, Data curation, Formal analysis, Investigation, Methodology, Validation, Writing – original draft. Z-GY: Conceptualization, Funding acquisition, Investigation, Methodology, Supervision, Writing – review & editing. JL: Formal analysis, Investigation, Supervision, Writing – review & editing.
