# Supplementary material for: CGRWDL: alignment-free phylogeny reconstruction method for viruses based on chaos game representation weighted by dynamical language model
Source: Front Microbiol. 2024 Mar 20;15:1339156. doi: 10.3389/fmicb.2024.1339156 (PMC10987876; doi:10.3389/fmicb.2024.1339156)
Supplement: Supplementary file 1 [file Table_1.DOCX]

Supplementary Material

**CGRWDL: Alignment-free phylogeny reconstruction method for viruses based on chaos game representation weighted by dynamical language model**

**Ting Wang^1,2^, Zu-Guo Yu^1,2,*^ , Jinyan Li^3^**

^1^National Center for Applied Mathematics in Hunan, Xiangtan University, Hunan 411105, China.

^2^Key Laboratory of Intelligent Computing and Information Processing of Ministry of Education, Xiangtan University, Hunan 411105, China

^3^School of Computer Science and Control Engineering, Shenzhen Institute of Advanced Technology, Shenzhen, Guangdong 518107, China

*** Correspondence:**

Zu-Guo Yu

yuzg@xtu.edu.cn

For example, if S=AGTCGTAACA is a DNA fragment, when *k*=2, ${CGR}_{a_{1}a_{2}\ldots a_{k}}\bar{x}$ and $\mathrm{CGR}_{a_{1}a_{2}\ldots a_{k}}\bar{y}$ are calculated as follows:

First, we mapped the above DNA fragments to the CGR map with coordinate positions represented as:

A: (0.25, 0.25);

AG: (0.625, 0.625); ${CGR}_{AG}$= (0.625, 0.625);

AGT: (0.8125, 0.3125); ${CGR}_{GT}$= (0.8125, 0.3125);

AGTC: (0.40625, 0.65625); ${CGR}_{TC}$= (0.40625, 0.65625);

AGTCG: (0.703125, 0.828125); ${CGR}_{CG}$= (0.703125, 0.828125);

AGTCGT: (0.8515625, 0.4140625); ${CGR}_{GT}$= (0.8515625, 0.4140625);

AGTCGTA: (0.42578125, 0.20703125); $\mathrm{CGR}_{\mathrm{TA}}$= (0.42578125, 0.20703125);

AGTCGTAA: (0.212890625, 0.103515625); ${CGR}_{AA}$ = (0.212890625, 0.103515625);

AGTCGTAAC: (0.1064453125, 0.5517578125); ${CGR}_{AC}$= (0.1064453125, 0.5517578125);

AGTCGTAACA: (0.05322265625, 0.27587890625);

${CGR}_{CA}$= (0.05322265625, 0.27587890625).

Then, we calculate the mean values and arrange them in *k*-mers alphabetical order as follows:

$${CGR}_{AA}\bar{x}=0.212890625; {CGR}_{AA}\bar{y}=0.103515625;$$

$${CGR}_{AC}\bar{x}=0.1064453125; {CGR}_{AC}\bar{y}=0.5517578125;$$

$${CGR}_{AG}\bar{x}=0.625; {CGR}_{AG}\bar{y}=0.625;$$

$${CGR}_{AT}\bar{x}=0; {CGR}_{AT}\bar{y}=0;$$

$${CGR}_{CA}\bar{x}=0.05322265625; {CGR}_{CA}\bar{y}=0.27587890625;$$

$${CGR}_{CC}\bar{x}=0; {CGR}_{CC}\bar{y}=0;$$

$${CGR}_{CG}\bar{x}=0.703125; {CGR}_{CG}\bar{y}=0.828125;$$

$${CGR}_{CT}\bar{x}=0; {CGR}_{CT}\bar{y}=0;$$

$${CGR}_{GA}\bar{x}=0; {CGR}_{GA}\bar{y}=0;$$

$${CGR}_{GC}\bar{x}=0; {CGR}_{GC}\bar{y}=0;$$

$${CGR}_{GG}\bar{x}=0; {CGR}_{GG}\bar{y}=0;$$

$${CGR}_{GT}\bar{x}=\frac{0.8125+0.8515625}{2}=0.83203125;$$

$${CGR}_{GT}\bar{y}=\frac{0.3125+4140625}{2}=0.36328125;$$

$${CGR}_{TA}\bar{x}=0.42578125; {CGR}_{TA}\bar{y}=0.20703125;$$

$${CGR}_{TC}\bar{x}=0.40625; {CGR}_{TC}\bar{y}=0.65625;$$

$${CGR}_{TG}\bar{x}=0; {CGR}_{TG}\bar{y}=0;$$

$${CGR}_{TT}\bar{x}=0; {CGR}_{TT}\bar{y}=0.$$

**Figure S1.** Phylogenetic tree of HCV complete protein-coding DNA sequences constructed by CGRWDL (k=8).

**Figure S2.** Phylogenetic tree of HBV complete DNA sequences constructed by CGRWDL (k=8).

**Figure S3**. Phylogenetic tree of HPV complete DNA sequences constructed by CGRWDL(k=8)

**Figure S4**. Phylogenetic tree of Dengue virus DNA sequences constructed by CGRWDL(k=8).

**Figure S5.** Phylogenetic tree of Ebola virus complete protein sequences constructed by CGRWDL(k=4)


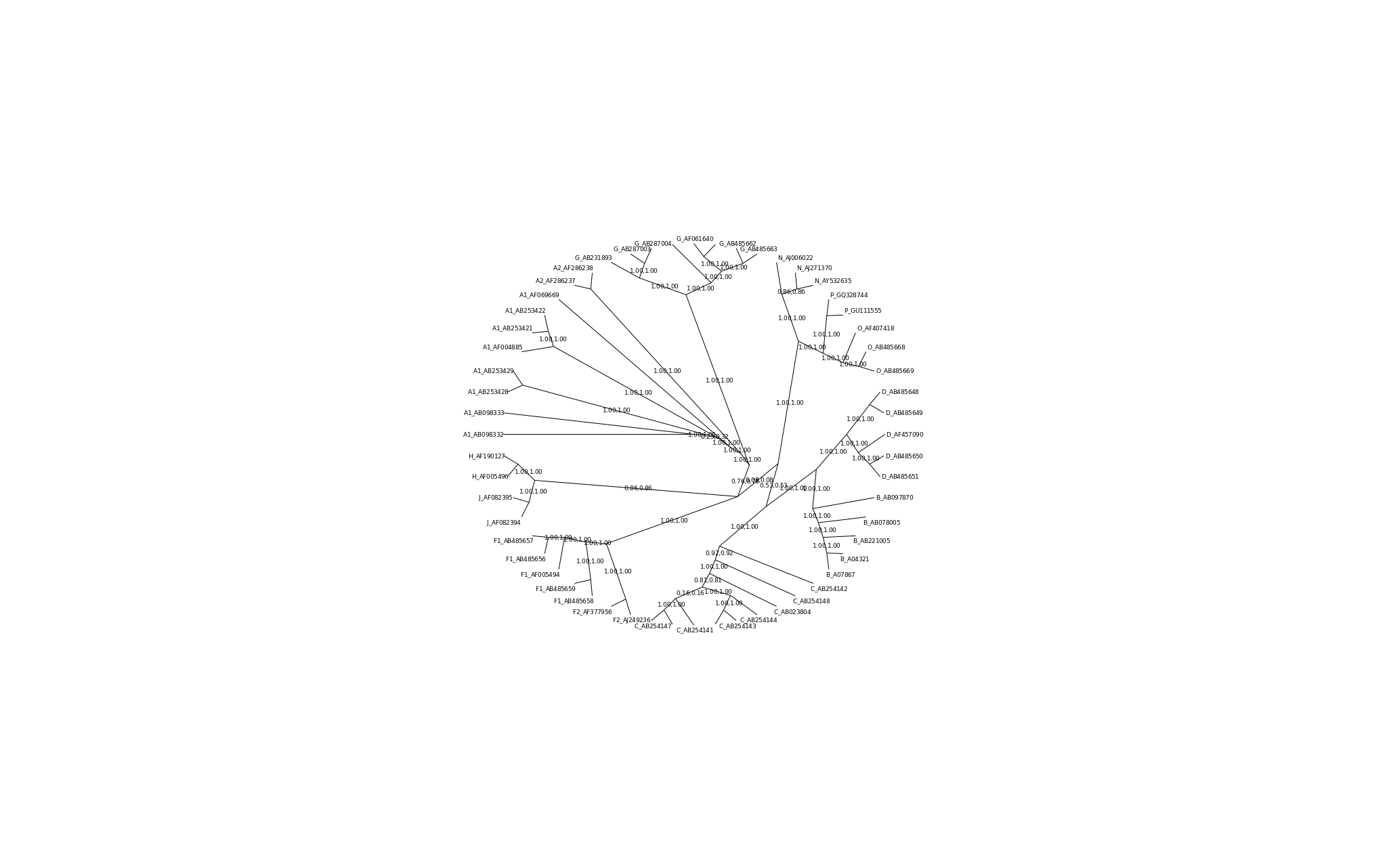


**Figure S6.**Inconsistency tree of HIV-1 sequences.


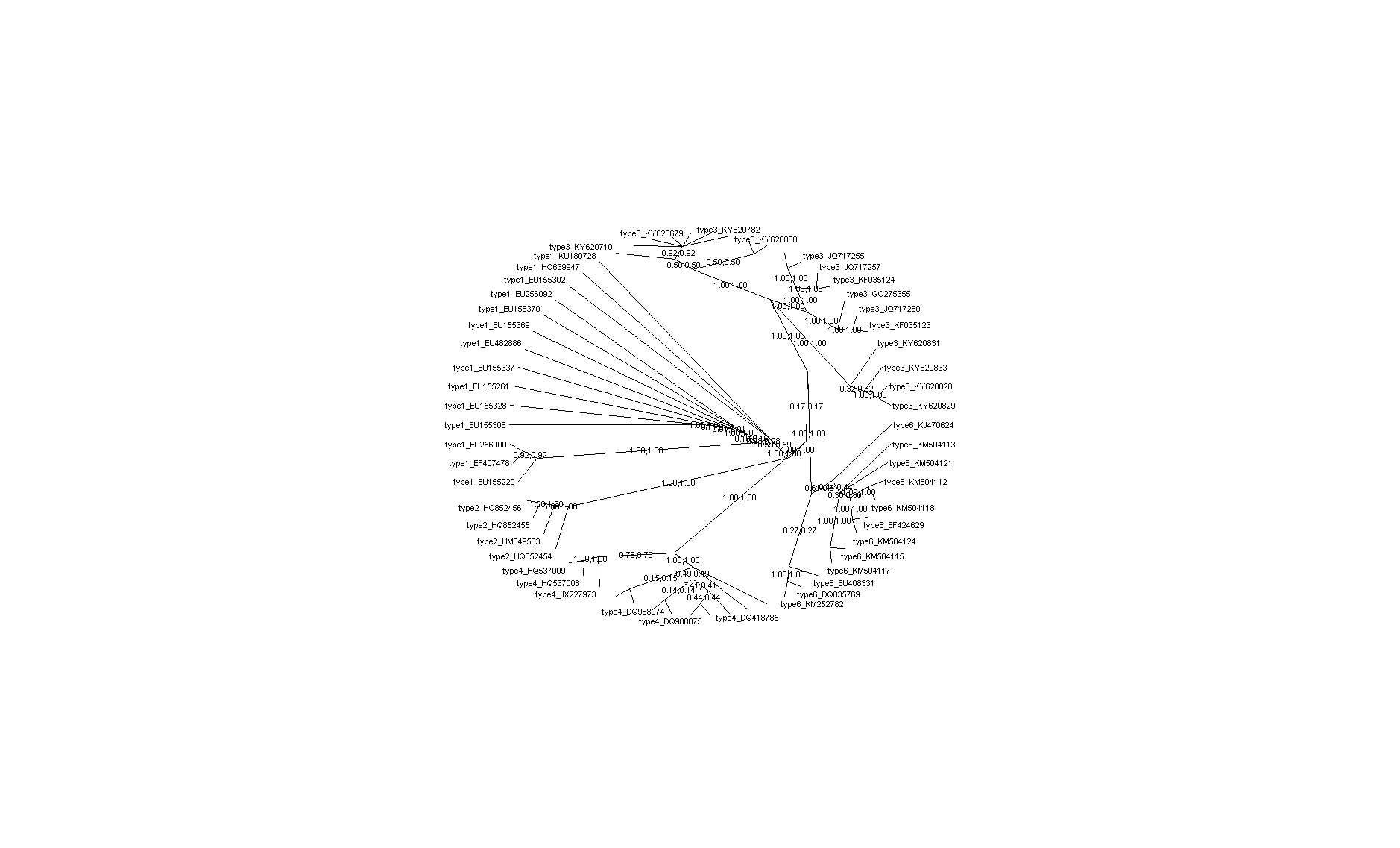


**Figure S7.** Inconsistency tree of HCV sequences


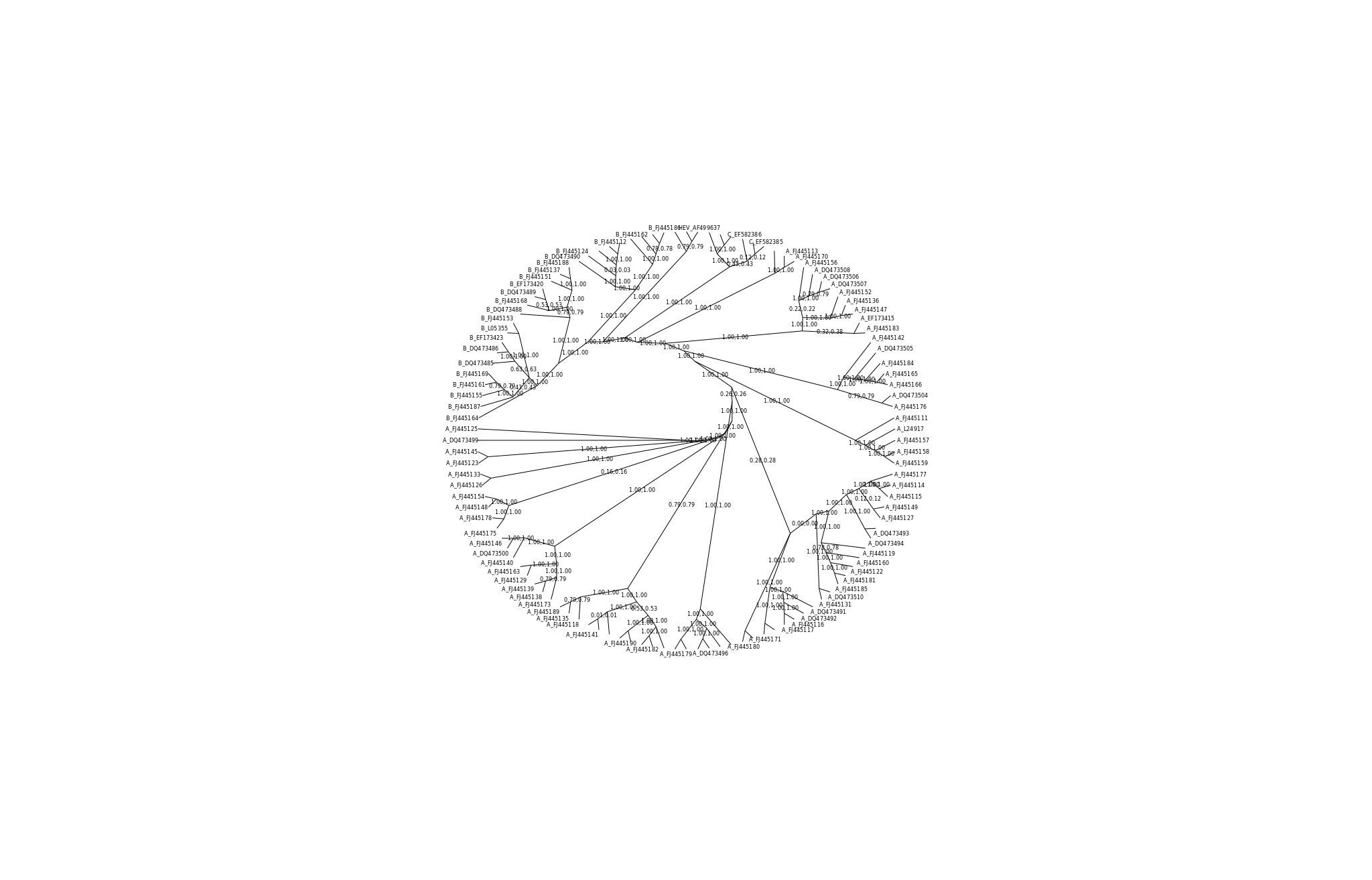


**Figure S8.** Inconsistency tree of HRV sequences.


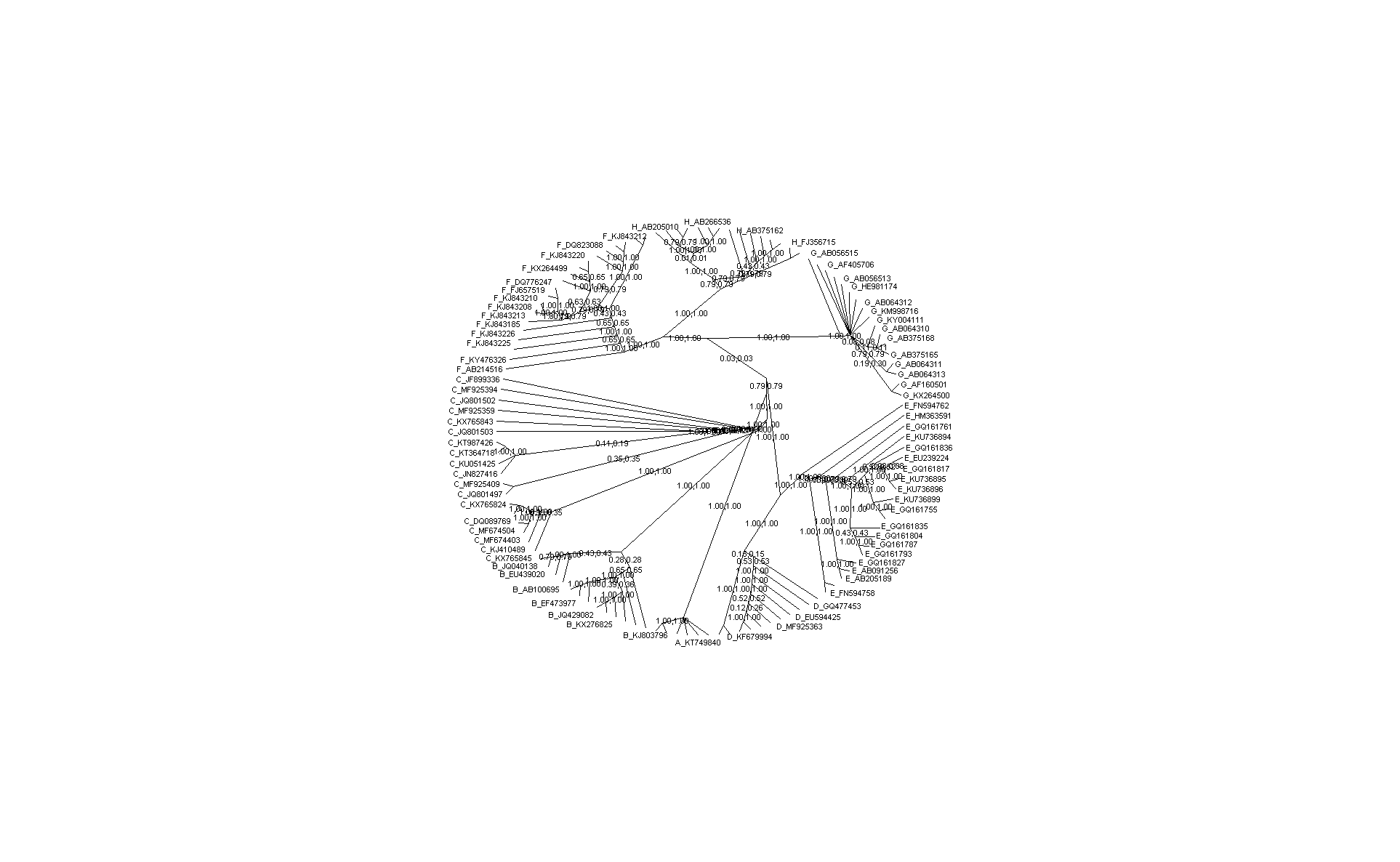


**Figure S9.** Inconsistency tree of HBV sequences


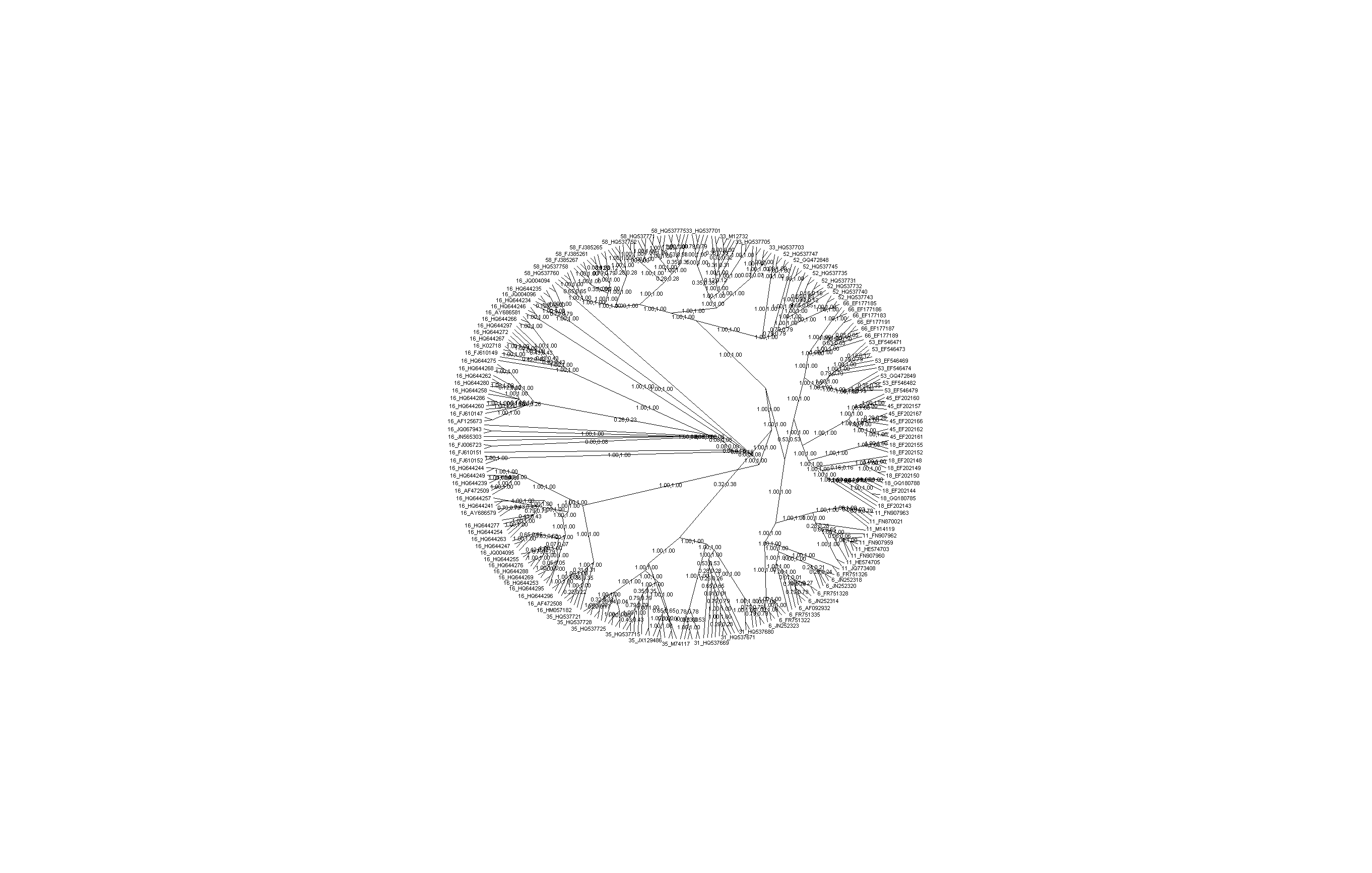


**Figure S10.** Inconsistency tree of HPV sequences


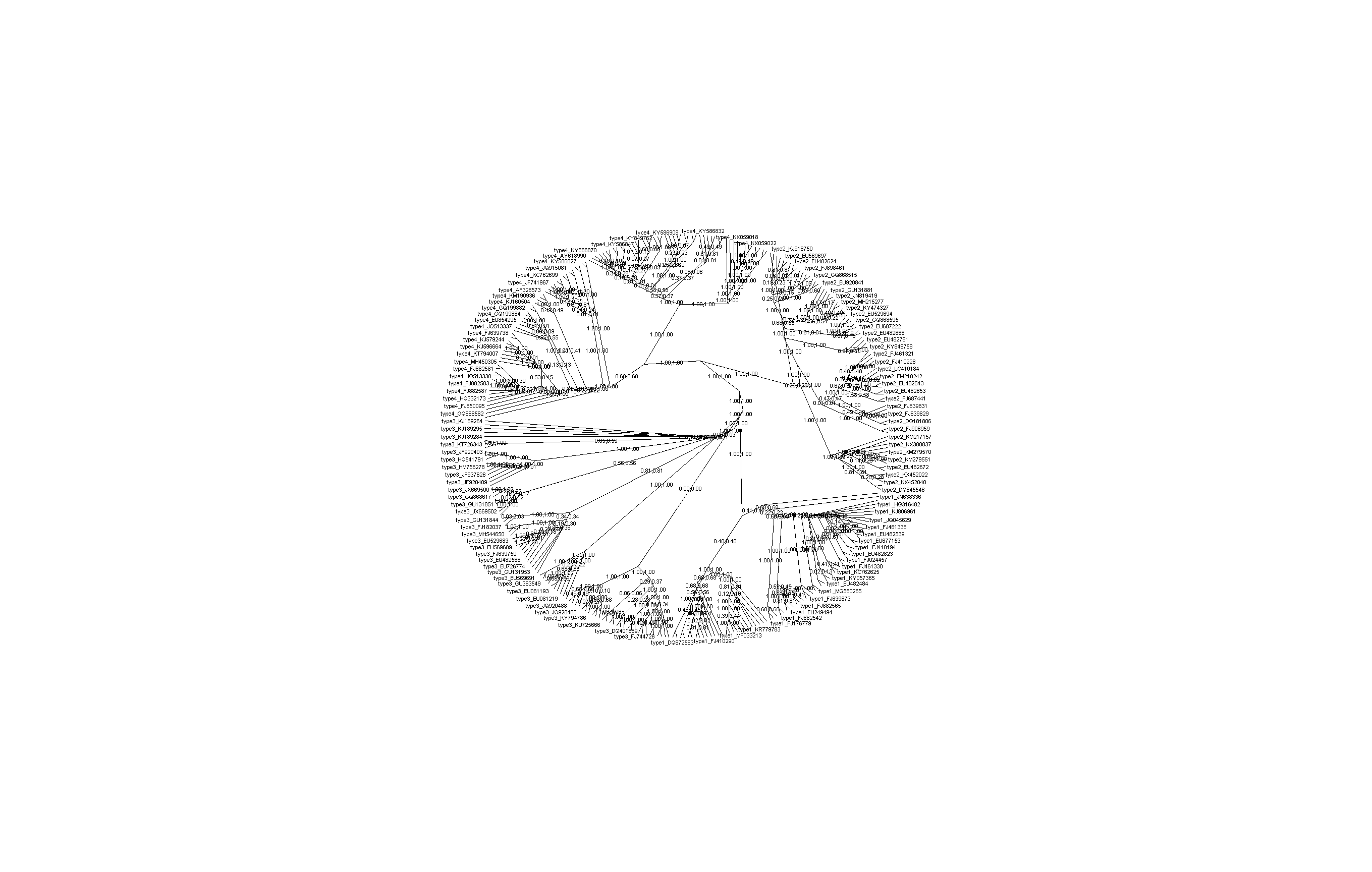


**Figure S11.** Inconsistency tree of Dengue virus sequences


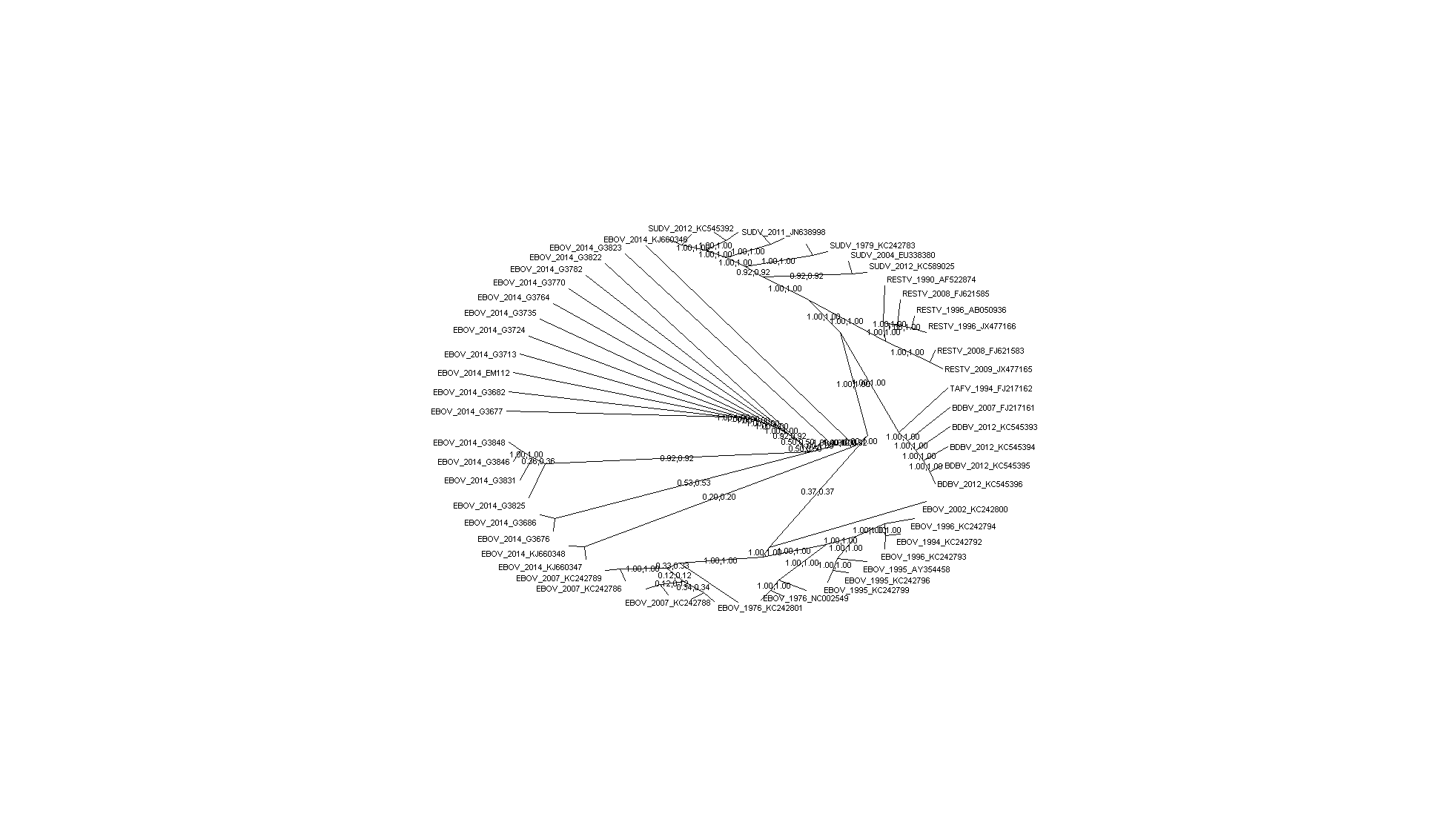


**Figure S12.** Inconsistency tree of Ebola virus sequences

**
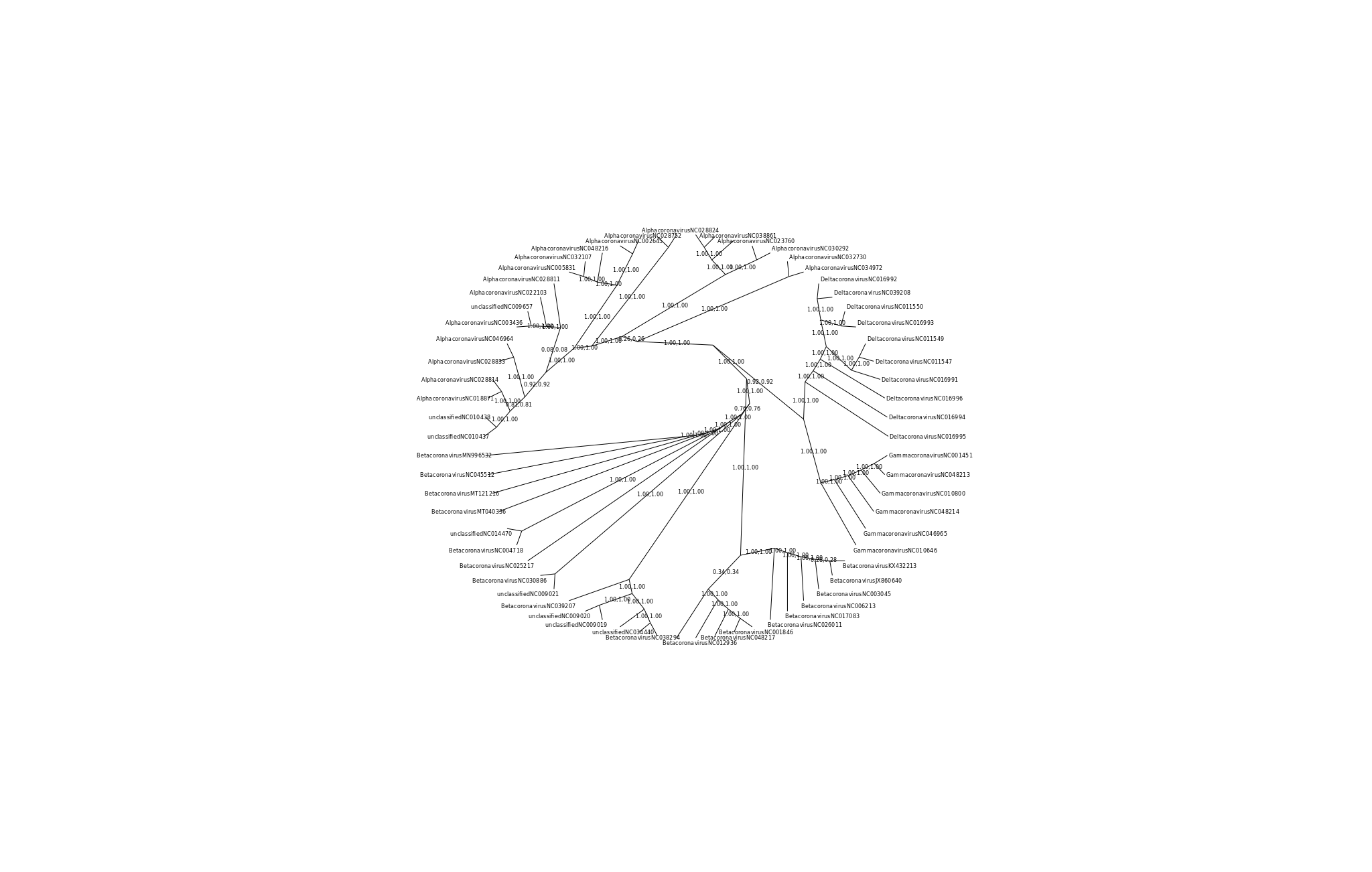
**

**Figure S13.** Inconsistency tree of Coronaviruses sequences.

**Table S1** The values of k-mer in seven methods

| Dataset | CGRWDL | DLTree | CVTree | FFP | d2 | KINN | FSWM |
| --- | --- | --- | --- | --- | --- | --- | --- |
| HIV | 8 | 8 | 8 | 6 | 8 | 6 | 8 |
| HCV | 8 | 9 | 9 | 6 | 8 | 6 | 9 |
| HBV | 8 | 8 | 8 | 6 | 8 | 6 | 9 |
| HPV | 9 | 9 | 7 | 6 | 8 | 6 | 8 |
| Dengueviruses | 8 | 9 | 9 | 9 | 9 | 6 | 9 |
| Coronavirus | 10 | 11 | 11 | 7 | 9 | 6 | 11 |
| Ebolaviruses(P) | 4 | 4 | 4 | 4 | 3 | 3 | / |
| HRV(P) | 4 | 4 | 4 | 4 | 3 | 3 | / |
